# Human Norovirus Aptamer Exhibits High Degree of Target Conformation-Dependent Binding Similar to That of Receptors and Discriminates Particle Functionality

**DOI:** 10.1128/mSphere.00298-16

**Published:** 2016-11-02

**Authors:** Matthew D. Moore, Benjamin G. Bobay, Brittany Mertens, Lee-Ann Jaykus

**Affiliations:** a Department of Food, Bioprocessing and Nutrition Sciences, North Carolina State University, Raleigh, North Carolina, USA; b Department of Molecular and Structural Biochemistry, North Carolina State University, Raleigh, North Carolina, USA; c Department of Chemical and Biomolecular Engineering, North Carolina State University, Raleigh, North Carolina, USA; University of Pittsburgh School of Medicine

**Keywords:** aptamer, GII.4, histo-blood group antigen, infectivity, noroviruses

## Abstract

Although two *in vitro* cultivation methods have been reported, discrimination of infectious human norovirus particles for study of viral inactivation is still a challenge, as both rely on reverse transcriptase quantitative PCR. Histo-blood group antigen (HBGA) binding assays serve as a proxy for estimation of infectious particles; however, they are costly and difficult to purify/modify. Some evidence suggests that certain nucleic acid aptamers only bind intact target proteins, thus displaying a high degree of conformation-dependent binding. The objective of this proof-of-concept study was to characterize the degree of conformation-dependent binding a human norovirus aptamer, M6-2, displayed with the capsid of the norovirus GII.4 Sydney (SYV) strain as a model. SYV capsids were exposed to heat, and aptamer, receptor (HBGA), and antibody binding was assessed. M6-2 and the receptor displayed similarly little target sequence-dependent binding (2.0% ± 1.3% and 0.5% ± 1.2% signal, respectively) compared to that of NS14 (26.4% ± 3.9%). The decay rates calculated with M6-2 and the receptor were also not statistically significantly different (*P* > 0.05), and dynamic light scattering and electron microscopy confirmed these observations. Ligand docking simulations revealed multiple distinct contacts of M6-2 in the N-terminal P1 and P2 domains of the viral capsid, with some residues close to receptor binding residues. These data suggest that single-stranded DNA aptamers like M6-2 display a high degree of target conformation-dependent binding. It is the first time nucleic acid aptamers have had this characteristic utilized and investigated to discern the infectivity status of viral particles, and the data suggest that other aptamers may show promise as valuable ligands in the study of other fastidious microorganisms.

**IMPORTANCE** Human noroviruses impose a considerable health burden globally. However, study of their inactivation is still challenging with currently reported cell culture models, as discrimination of infectious viral particles is still difficult. Traditionally, the ability of particles to bind putative carbohydrate receptors is conducted as a proxy for infectivity, but these receptors are inconsistent, expensive, and hard to purify/modify. We report a hitherto unexplored property of a different type of ligand, a nucleic acid aptamer, to mimic receptor binding behavior and assess capsid functionality for a selected strain of norovirus. These emerging ligands are cheaper, more stable, and easily synthesized/modified. The previously unutilized characteristic reported here demonstrates the fundamental potential of aptamers to serve as valuable, accessible tools for any microorganism that is difficult to cultivate/study. Therefore, this novel concept suggests a new use for aptamers that is of great value to the microbiological community—specifically that involving fastidious microbes.

## Abstract (precis)

Human noroviruses impose a considerable health burden globally. However, study of their inactivation is still challenging with currently reported cell culture models, as discrimination of infectious viral particles is still difficult. Traditionally, the ability of particles to bind putative carbohydrate receptors is conducted as a proxy for infectivity, but these receptors are inconsistent, expensive, and hard to purify/modify. We report a hitherto unexplored property of a different type of ligand, a nucleic acid aptamer, to mimic receptor binding behavior and assess capsid functionality for a selected strain of norovirus. These emerging ligands are cheaper, more stable, and easily synthesized/modified. The previously unutilized characteristic reported here demonstrates the fundamental potential of aptamers to serve as valuable, accessible tools for any microorganism that is difficult to cultivate/study. Therefore, this novel concept suggests a new use for aptamers that is of great value to the microbiological community—specifically that involving fastidious microbes.

## INTRODUCTION

Human norovirus is the leading cause of acute viral gastroenteritis, responsible for about 125 million cases and a disease burden of 2.5 million disability-adjusted life years lost annually globally (1, 2). After decades of effort, two *in vitro* cultivation systems for human norovirus have recently been reported (3, 4). One system suggests that enteric bacteria are required for viral infection; however, it has been difficult to replicate the system outside the lab in which it has been observed (4, 5). Another study using human enteroids has recently been reported and replicated in multiple labs (3). Although major breakthroughs, these systems still utilize reverse transcriptase quantitative PCR (RT-qPCR) for quantification and do not produce enough virus for proper sensitivity in the study of viral inactivation agents. A number of cultivable surrogate viruses have been used to estimate the behavior of human norovirus relative to environmental stresses or inactivation methods. However, none of these surrogates is ideal (6). RT-qPCR remains the most common method of detecting and quantifying noroviruses (including in the culture methods mentioned above), but because it relies on the amplification of a small segment of the viral genome, it cannot estimate the number of infectious particles. This is because amplification results will include (i) intact genomes from infectious particles, (ii) partially degraded or fatally mutated genomes from intact and degraded particles, (iii) free norovirus RNA not associated with a capsid, and (iv) genomes from defected/damaged viral particles. Therefore, RT-qPCR frequently overestimates viral infectivity. This is problematic, as overestimation of infectivity confounds inactivation data, resulting in inaccurate estimates that may suggest that a method performs poorly when, in fact, it is quite efficacious.

Multiple upstream *in vitro* methods have been developed to facilitate the discrimination of virus infectivity status. Generally, approaches to do this focus on assessing viral genome integrity and/or capsid integrity/functionality (7). One method that is widely used is viral receptor binding, most commonly done with histo-blood group antigens (HBGAs). HBGA (or porcine gastric mucin [PGM], which contains some HBGAs) binding has performed well in comparison with the plaque assay when murine norovirus and Tulane virus surrogates were subjected to a variety of physical and chemical stresses (8–10). In the most compelling work yet, GI.1 Norwalk virus inactivation estimated by a combined PGM binding RT-qPCR method compared favorably to results from a parallel human challenge study evaluating the efficacy of high-pressure processing on norovirus in oysters (11, 12).

However, purified HBGAs are not ideal human norovirus binding ligands. These carbohydrate receptors are fairly costly, may require purification from animals, and cannot be easily functionally modified with chemical groups or synthesized. Because human norovirus binding is strain specific, no HBGA broadly reacts with all human norovirus strains, and some human noroviruses do not bind any HBGAs (13, 14). Although cheaper than synthetic HBGAs, PGM is not easily chemically modified, has potential additional components that can confound assay results, is derived from animals, may vary in HBGA quality/proportion, and is also not broadly reactive to human noroviruses. Antibodies also may have potential application for infectivity discrimination, but with several of the same limitations (15–17). One monoclonal antibody, NS14, has shown promise as a broadly reactive reagent for the GII genogroup of noroviruses (16, 18) and is thus an improvement on HBGAs. Aptamer M6-2, generated against a GII.4 human norovirus strain, has been found to react to GI and GII human noroviruses, binding to all of the noroviruses tested in a panel of 14 human norovirus strains (19). We hypothesized that nucleic acid aptamers may be an alternative ligand for use in infectivity discrimination. They offer the advantages of low cost; ease of synthesis, purification, and functional modification; and high stability. Multiple aptamers have been generated for human norovirus binding, some of which are broadly reactive (19–22). Evidence has been presented that aptamers tend to bind their targets with multiple different discriminatory interactions in unique ways (23), and as a consequence, instances have been reported in which aptamers did not bind denatured target proteins (24–26). The purpose of this study was to determine the utility of aptamers for use as alternative ligands in estimating the capsid functionality of heat-treated human norovirus in comparison with HBGAs and antibodies, by using the most recent strain of epidemic human norovirus as a model.

## RESULTS

In this work, the target structure-dependent binding ability of a nucleic acid aptamer, M6-2, was compared to that of two more traditional ligands, an HBGA and a monoclonal antibody, by using plate-based binding assays. SYV virus-like particles (VLPs) subjected to different heat treatments were applied to plates, and binding of the ligands was assessed. To confirm binding assay observations, heated SYV VLPs were also analyzed by dynamic light scattering (DLS) and transmission electron microscopy (TEM).

### Plate binding and morphological data.

**FIG 1:**
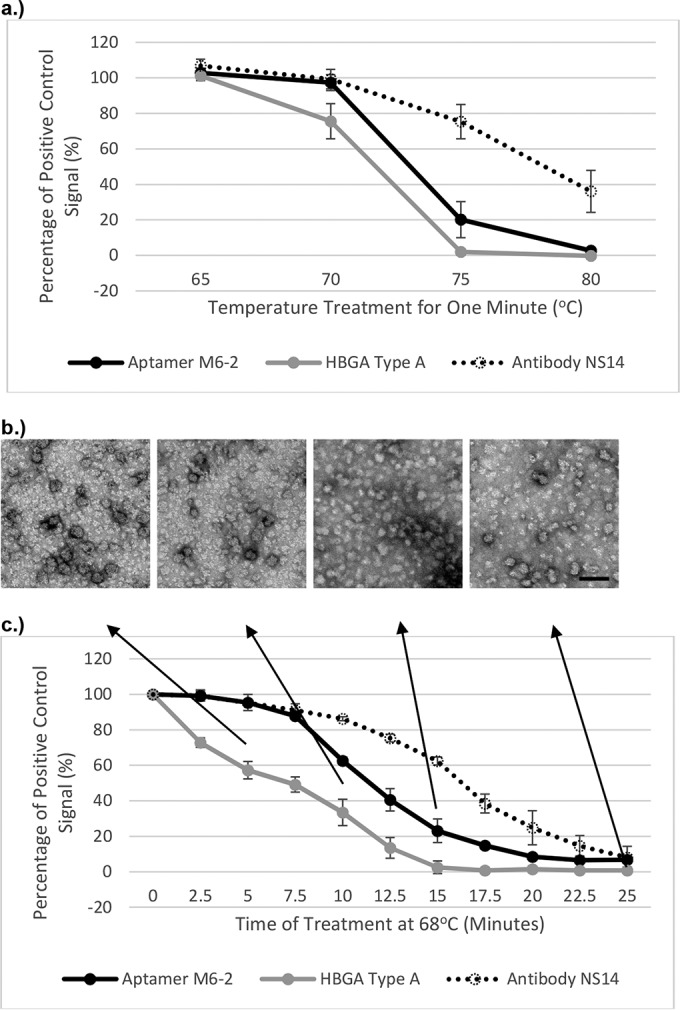
Conformation-dependent binding of three different ligands to SYV VLPs treated at selected time-temperature combinations. (a) Signals of aptamer M6-2, HBGA type A, and antibody NS14 binding to SYV VLPs treated at various temperature for 1 min are reported as percentages of signals of binding to untreated VLPs. (b) TEM images of SYV VLPs treated at 68°C for 5, 10, 15, or 25 min. The scale bar represents 100 nm. (c) Signals of aptamer M6-2, HBGA type A, and antibody NS14 binding to SYV VLPs treated at 68°C for various times are reported as percentages of signals of binding to untreated SYV VLPs.

The structure-dependent binding behavior of broadly reactive human norovirus aptamer M6-2, a biotinylated synthetic HBGA, and monoclonal antibody NS14 was investigated by using VLPs of the most recent pandemic norovirus strain, GII.4 Sydney, as a model in this proof-of-concept study. The behavior of broadly reactive GII antibody NS14 (16) was included to compare the behavior of more traditional ligands with HBGAs and the aptamer. Overall, aptamer binding more closely correlated with HBGA binding than it did with antibody NS14. However, at each corresponding temperature for 1 min, the HBGA binding signal level was lower than the aptamer M6-2 signal level (Fig. 1a). With the HBGA results used as the “gold standard,” aptamer M6-2 binding slightly overestimated capsid functionality. Comparatively speaking, at each temperature, the antibody binding signal was the highest observed, indicating a higher degree of overestimation of capsid functionality compared to HBGA. Unlike aptamer M6-2, the NS14 binding signal trend did not generally correspond to that of the HBGA signal. The greatest difference among the three ligand signals was seen at 75°C, where the HBGA binding signal was observed at 1.9% ± 1.6% signal and the aptamer and antibody signals were observed at 20.1% ± 10.1% and 75.3% ± 9.7%, respectively. Complete loss of binding signal after treatment at 80°C for 1 min was observed for HBGA and aptamer M6-2, but the NS14 signal level remained significantly higher (*P* < 0.05) at 36.1% ± 11.8%. TEM images of SYV VLPs at the different temperatures corresponded to the observed signal loss, with some morphological changes consistent with viral capsid denaturation, such as increased opacity, disruption of spherical structure, and increased aggregation/clumping visible at higher temperatures (see Fig. S1 in the supplemental material).

10.1128/mSphere.00298-16.1 Figure S1  Electron microscopy of SYV VLPs treated at different temperatures for 1 min. SYV VLPs were treated for 1 min at various temperatures and immediately cooled to 4°C. From left to right: 65, 70, 75, and 80°C. The cooled solutions were then applied to grids, stained, and viewed in a JEOL transmission electron microscope at 80 kV at a magnification of ×50,000. Download Figure S1, PDF file, 0.6 MB. Copyright © 2016 Moore et al. 2016 Moore et al. This content is distributed under the terms of the Creative Commons Attribution 4.0 International license.

Longer periods at temperatures of 65°C and 68°C were chosen to further investigate trends observed with the initial 1-min exposure experiments. At 65°C, nearly complete conformation-dependent signal loss was observed for all three ligands after 100 min (see Fig. S2 in the supplemental material). Interestingly, aptamer and antibody ligands overestimated the VLP binding signal level relative to HBGA from 0 to 37.5 min and then a notable separation of signal was observed among all three ligands. At these later times, the aptamer signal loss trend generally mimicked that of the HBGA, whereas antibody NS14 was again notably different. This is supported by the calculated binding signal exponential decay rates at 65°C, as the aptamer (−2.5% ± 0.2% signal/min) and HBGA (−2.5% ± 0.7% signal/min) signal decay rates were not significantly different (*P* > 0.05), but the NS14 signal decay rate (−1.4% ± 0.3% signal/min) was significantly different (*P* < 0.01) from that of HBGA. Similar results were observed with VLPs treated at 68°C, for which the exponential decay rates of M6-2 and HBGA were not statistically significantly different (*P* > 0.05), but that of NS14 was significantly different (*P* < 0.001) (Table 1). Morphological changes in the capsids at 68°C observed by TEM were first seen after 15 min, as capsids became opaque and deformed and began to aggregate. However, even after the receptor binding signal was lost, a few intact capsids still remained.

10.1128/mSphere.00298-16.2 Figure S2  Conformation-dependent binding of three different ligands to SYV VLPs treated at 65°C. Signals of aptamer M6-2, HBGA type A, and antibody NS14 binding to SYV VLPs treated at 65°C for various times are shown. Signals are reported as percentages of those obtained with untreated VLPs. Download Figure S2, PDF file, 0.3 MB. Copyright © 2016 Moore et al. 2016 Moore et al. This content is distributed under the terms of the Creative Commons Attribution 4.0 International license.

**TABLE 1:** Exponential decay rates of SYV calculated with different ligands^a^

Ligand	Exponential decay rate (% signal/min) at:	
	65°C	68°C
HBGA	−2.50 ± 0.24†	−13.30 ± 1.15§
M6-2	−2.47 ± 0.66†‡	−13.18 ± 0.47§
NS14	−1.36 ± 0.27‡	−9.19 ± 2.06¶

a SYV VLPs were heated and applied to polystyrene plates for analysis of binding with each ligand. The percentage of the signal was calculated by taking the absorbance for each treatment as a percentage of that of an untreated control. The signal of binding to completely denatured VLPs was subtracted from all absorbance values. The rates shown are percentage of the signal per minute. Values followed by dissimilar symbols are statistically significantly different.

**FIG 2:**
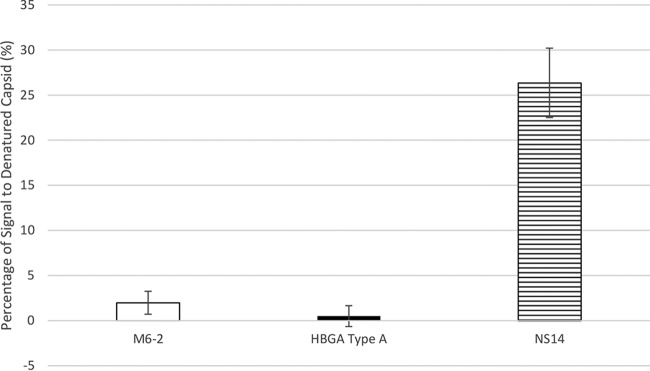
Apparent percentages of sequence-dependent binding of three human norovirus ligands. Normalized signal for aptamer M6-2, HBGA type A, and antibody NS14 binding to completely denatured SYV VLPs (treated at 80°C for 5 min) as percentages of signal for binding to untreated SYV VLPs.

To estimate the degree of sequence-dependent binding, the signal obtained with completely denatured SYV VLPs was determined as a percentage of that obtained with untreated SYV VLPs (Fig. 2). Aptamer M6-2 (2.0% ± 1.3%) and HBGA (0.5% ± 1.2%) displayed very small proportions of binding to completely denatured SYV VLP that were not statistically significantly different (*P* > 0.05). However, antibody NS14 displayed a significantly greater (*P* < 0.05) degree of binding to completely denatured SYV VLPs (26.4% ± 3.9%). This implies that the binding of both aptamer M6-2 and HBGA to SYV VLPs strongly depends on the maintenance of capsid secondary, tertiary, and quaternary structures and further supports the above-described observations of similar aptamer and HBGA heat-treated SYV VLP binding behaviors.

### DLS observations.

**FIG 3:**
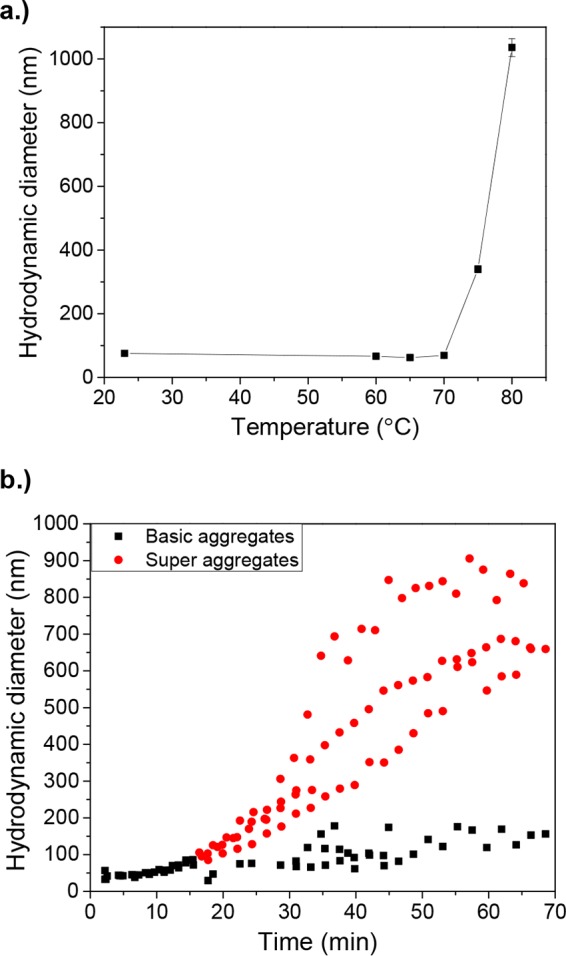
DLS results for SYV VLPs treated at different temperatures and/or times. (a) Hydrodynamic diameter of SYV VLPs treated at different temperatures for 1 min. Error bars represent the standard error of three measurements taken for each of two samples at each temperature. (b) Kinetics of SYV VLP aggregation for three samples treated at 68°C for various times.

DLS data supported the binding results, as a major shift in aggregation occurred when the 70°C and 75°C treatments were compared after 1 min (Fig. 3a). This very closely corresponds to the ~80% drop in the binding signal level observed with aptamer M6-2 and HBGA (Fig. 1a). Thermal quenching after 1-min treatments did not result in a breakdown of aggregates, indicating irreversible aggregation. At 68°C, aggregation occurred after 10 min, with a large increase in diameter by 15 min (Fig. 3b). The Zetasizer software distinguished the formation of superaggregates at about 15 min, indicating that significant changes in capsid protein morphology have occurred to allow the formation of aggregates. These results generally corresponded to the observed loss of binding but occurred slightly after a large degree of HBGA binding had been lost (~40%), which also supports the hypothesis that slight changes in higher-order protein structure can notably reduce HBGA binding before morphological changes are observed. Interestingly, because of the slightly lower sensitivity to changes in capsid structure due to heat, aptamer M6-2 binding results closely match those observed with DLS, as hardly any signal was lost after 5 min at 68°C, but ~80% of the signal was lost after 15 min, when superaggregation began to occur and aggregation increased notably (Fig. 3).

### Aptamer structural prediction and molecular docking to SYV capsid protein. (i) Construction of aptamer M6-2 and SYV capsid protein structures.

**FIG 4:**
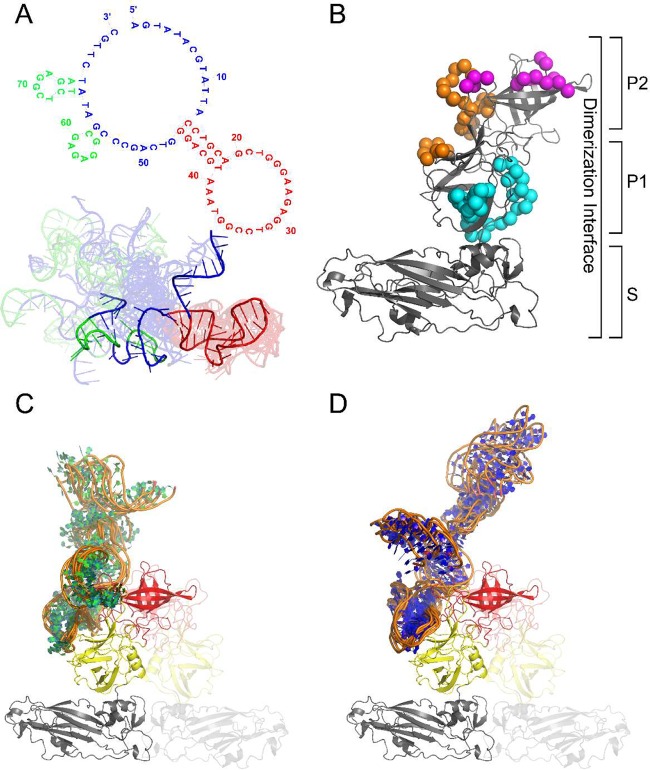
Molecular docking of M6-2 to SYV VP1. M6-2 secondary and tertiary structures used in the molecular docking process are shown in panel A with different motifs colored accordingly. The color scheme of the secondary and tertiary structures is consistent. The structural ensemble for the M6-2 ssDNA aptamer is shown in cartoon format with the average structure highlighted in darker colors and the full ensemble used in the molecular docking faded in the background to visually provide a sense of the conformational flexibility of the ssDNA aptamer. Panel B conveys the potential novel interaction surface of M6-2 (orange spheres), HBGA (magenta spheres), and antibody NS14 (cyan spheres). Panels C and D show the HADDOCK-docked M6-2 ssDNA aptamer clusters to SYV VP1. The dimeric partner of SYV VP1 is shown in faded cartoon format to hightlight the dimerization interface. Each individual domain of SYV VP1 is colored differently, i.e., gray, yellow, and red for the S, P1, and P2 domains, respectively.

M6-2 aptamer Protein Data Bank (PDB) file construction showed secondary structure similar to that of the Mfold prediction, and this secondary structure was retained throughout the full 100-ns molecular dynamics (MD) simulation (color coded for visualization in Fig. 4a). The SYV dimeric VP1 capsid structure was successfully generated, resulting in good bond and angle geometries and with >95% of the residues in generously allowed or better Ramachandran phi/psi dihedral space. The resulting model of SYV overlaid with a Cα root mean square deviation (RMSD) of 3.69 Å to the template structure (PDB code 1IHM; see Fig. S4 in the supplemental material) (27), with the largest RMSD owned by the P2 domain.

### (ii) Docking simulation of aptamer M6-2 to SYV capsid protein.

The HADDOCK molecular docking approach was employed to develop a structural model of the interaction between M6-2 and SYV VP1. This was approached as a blind docking study of the two entities with the following caveats. (i) Active residues (residues directly involved in the interaction) within SYV VP1 were defined as residues with >50% solvent accessibility (calculated by Naccess) (28). (ii) No residues within the S domain of SYV VP1 were defined as active, as the aptamer is known not to bind to the S domain. (iii) No residues within the dimerization interface of SYV VP1 within the P1/P2 domain were defined as active residues, as it is unlikely that the M6-2 aptamer disrupts this large dimerization interface. (iv) Active bases within the DNA aptamer were defined as bases 14 to 45, while passive residues were defined as bases 1 to 13 and 46 to 81 (Fig. 4A).

The molecular docking between M6-2 and SYV VP1 resulted in four clusters of docked structures based on a maximum Cα and P (backbone phosphate) RMSD of 7.5 Å and a minimum of five structures per cluster. Clusters 1 and 2 resulted in eight and six, respectively, clustered structures in an orientation that provided the maximum buried surface area while positioned in orientations that would not be sterically hindered in an assembled capsid (significant contact with the S domain) (Fig. 4C and D; see Fig. S5A and B in the supplemental material). Clusters 3 and 4 resulted in an aptamer orientation that would be sterically hindered in an assembled capsid (contact with the S domain and potential inaccessibility due to surrounding dimers of VP1 in an assembled capsid) (see Fig. S5C and D). The Cα RMSD between unbound SYV VP1 and the lowest-energy structure from cluster 1 of bound SYV VP1 was approximately 1.6 Å over residues within the P1 and P2 domains. This suggests that SYV VP1 undergoes a minor conformational change when it binds to M6-2, which has been previously observed with other aptamers (23). The interface between M6-2 and SYV VP1 revealed an extended network of hydrogen bonds and van der Waals interactions. On average, cluster 1 had 21 hydrogen bonds and 20 van der Waals interactions per structure, while cluster 2 had 8 hydrogen bonds and 20 van der Waals interactions per structure. Both clusters resulted in similar buried surface areas of ~2,800 Å^2^. These interactions are highlighted in Fig. 4B as orange spheres. The docking simulation predictions were confirmed by mutational analysis of aptamer M6-2. Native M6-2 exhibited binding, considered a VLP/no-VLP ratio of >2.0 per convention (8, 19, 21), for all of the aptamer concentrations tested, except at 0.001 µM. None of the other mutated aptamers displayed binding at any concentration, confirming docking simulation predictions.

## DISCUSSION

Although two groundbreaking studies showing replication of human norovirus in mammalian cells have recently been reported (3, 4), neither is yet ideal for a proper study of human norovirus inactivation agents. This is due to multiple aspects; i.e., the level of virus produced is not sufficient to allow the sensitivity to observe or model viral reduction to the level of regulatory significance, both methods still rely on RT-qPCR, and both methods utilize addition of potentially confounding agents (enteric bacteria or bile extract). Previously, in the absence of a widely available mammalian cell culture or animal model, ligand binding has served as a popular proxy for discrimination of infectious from noninfectious norovirus particles. Four studies specifically utilizing ligand binding have shown mostly favorable results compared to infectivity assay results for human norovirus and its cultivable surrogates. For example, Hirneisen and Kniel (8) found PGM binding to correlate well with plaque assay results for murine norovirus exposed to different heat treatments. Another study utilizing PGM binding for Tulane virus exposed to ethanol, chlorine, and heat showed reductions similar to those observed by 50% tissue culture infective dose determination (10). When PGM was used to evaluate the inactivation of GI.1 Norwalk virus by high-pressure processing, reductions corresponded to those observed in a human feeding study with the same virus (11, 12). On the other hand, Li and Chen (9) found that their PGM binding assay correlated with infectivity assay results for Tulane virus and murine norovirus treated with high pressure at a ≤2-log_10_ reduction, but the infectious virus titer was overestimated at higher degrees of inactivation. Another recent report found that PGM binding was more conservative than other *in vitro* infectivity assays for Tulane virus (29).

On the basis of the body of evidence supporting the use of HBGA-based binding assays as a potential way to exclude noninfectious particles prior to amplification by RT-qPCR, this study focused on comparing the binding behavior of single-stranded DNA (ssDNA) aptamers to HBGA and antibody. All three binding assays (HBGA, antibody, and aptamer) were derived from one another (21, 30). All three also utilized horseradish peroxidase (HRP) as a reporter and were developed with the same substrate system. All signals were endpoint readings presented as negative-control (denatured VLPs)-adjusted percentages of positive controls, greatly minimizing the likelihood that assay artifacts caused the observed differences in ligand performance. Although NS14 displayed the highest signal for denatured or partially denatured capsids, the only major difference between enzyme-linked immunosorbent assay (ELISA) and enzyme-linked aptamer sorbent assay (ELASA)/HBGA binding assays was the use of a streptavidin-HRP conjugate versus a secondary antibody-HRP conjugate. If anything, this would result in a reduced signal, as the antibody-antigen interaction is much weaker than the biotin-streptavidin one. On the basis of the assay similarities, we are confident that the results presented here are directly comparable. However, it must be noted that although these assays allow for relative comparisons, absolute quantification (i.e., log_10_ reductions) was not possible because of the generally low protein concentrations used and their absolute relationship to assembled human norovirus VLPs. In this study, multiple preparations of SYV VLPs were used, and this may account for some of the variation seen within and between assays. VLPs were prepared and purified by widely utilized methods (16, 31, 32), reducing the chance that effects or quality of the VLP preparation had any deleterious effect on the observations reported here. Further, the general purity observed with untreated VLP preparations by DLS (below) and seen in TEM further suggests that preparation had little effect on the results.

While aptamer M6-2 appeared to slightly overestimate capsid functionality on the basis of signal relative to a positive control compared to HBGA, its curve generally mimicked the shape and apparent exponential decay rate of capsid conformational loss shown for HBGA. This was not the case for antibody NS14, which overestimated capsid functionality relative to the findings for HBGA and underestimated the decay rate (Table 1). Differences between M6-2 and HBGA are likely due to the fact that the different ligands bind to close but different epitopes on the norovirus capsid, and these epitopes maintain their conformations under slightly different temperature stringencies. NS14 binds a completely different subdomain of the capsid and displays a reasonable degree of target conformation-dependent binding but has a considerable portion of target sequence-dependent binding. As shown in Fig. 2, M6-2 and HBGA display very little target sequence-dependent binding, as indicated by the apparent percentage of signal (<5%) due to binding of the completely denatured capsid compared to NS14 (26%). A high degree of conformational dependence has been demonstrated by using heat-denatured Norwalk virus VLPs and synthetic HBGA (33) and has been observed for other treatments (30), as HBGAs and receptor binding have been found to approximate capsid functionality and infectivity for heat- and high-pressure-treated virus (8, 11). Similar observations of aptamer dependence on nondenatured target protein have been reported (23), and several studies have shown that aptamer binding to proteins is highly dependent on the specific presentation of multiple distant residues of the target protein, e.g., for the well-studied thrombin aptamer (34–37). Ligand docking data predict such multiple interacting residues for M6-2 binding to the SYV capsid protein (Fig. 4). These factors would explain the observed sensitivity of aptamer M6-2 binding in a manner similar to HBGAs in heat-treated VLPs. The contributions of the length of the aptamer and the size of the target have not been evaluated; but one may speculate that the relatively longer sequence of aptamer M6-2 (80 nucleotides) and the larger size of its protein target (human norovirus P domain; 58 kDa) favor such a degree of conformation-dependent binding. This is because the larger size of both the aptamer and the target allows for multiple distant contacts to occur between the two, which can be especially critical for binding because of the limited diversity of pairing interactions that can occur with a nucleic acid as opposed to the larger diversity of protein properties (i.e., hydrophobic and positively charged residues). Although generally thought of as a limitation, this lack of diversity may function to limit the number of possible binding residues of M6-2 and account for the high dependence on target conformation, as interacting residues need to be oriented in a specific way. On the other hand, the residues interacting with M6-2 are not the same as those of HBGA, possibly explaining the slight overestimation of intact capsid observed for M6-2.

In this study, TEM images of capsid integrity roughly correlated with loss of HBGA binding when the temperature was varied at a given time, but capsids appeared to retain their structure after loss of receptor binding when the time was varied at a given temperature. This is consistent with the observations of other studies (30, 38) and is somewhat expected, given the highly conformation-dependent nature of HBGA binding discussed above. This phenomenon suggests that evaluation of aptamer or HBGA binding as a measure of infectivity would be more conservative than other methods used to discriminate infectious from noninfectious virus, i.e., the RNase protection assay. Specifically, pretreatment of samples with RNase prior to RT-qPCR is not likely to account for particles that remain intact but have lost HBGA or aptamer binding functionality. It has generally been observed that RNase pretreatment underestimates reductions observed by plaque assay (39, 40). Specifically, one study that directly compared a receptor (cell binding)-based assay to RNase pretreatment for murine norovirus subjected to heat found that cell binding more closely predicted viral reduction than did RNase treatment prior to RT-qPCR (41). Similarly, another study observing high-pressure inactivation of murine norovirus found that reductions when using RNase pretreatment did not correlate with cell culture (42). Other more promising results have been reported for such methods with feline calicivirus when modeling was applied (43); however, feline calicivirus is a *Vesivirus* and not as closely related to human noroviruses as murine norovirus.

DLS data agreed with binding observations, as VLPs began to aggregate as they denatured. Capsid proteins denature over an initial time period and form small aggregates that are labeled “basic aggregates.” At a time point characteristic of the protein, temperature, and protein concentration, these basic aggregates begin to combine to form larger clusters that are labeled “superaggregates” (44). Heat-induced aggregation occurs as a result of the exposure of hydrophobic residues upon protein unfolding, so the time at which superaggregate formation begins indicates that significant conformational changes to the capsid protein have taken place. Our results indicate that this superaggregation occurred as HBGA and aptamer binding signals were lost. This observation suggests that HBGA and aptamer binding is very sensitive to even slight degrees of protein denaturation.

**FIG 5:**
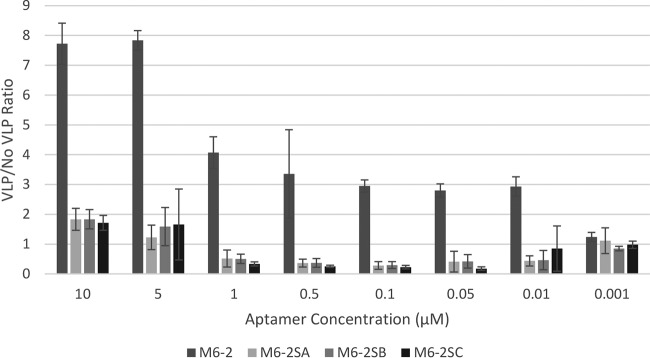
Binding analysis of predicted aptamer M6-2 binding region. Binding ELASAs of native aptamer M6-2 compared to three versions of M6-2 with the suspected binding region sequence mutated (Table 2; see Fig. S3 in the supplemental material) were conducted. Different concentrations of the aptamers were evaluated for binding to SYV VLPs. Ratios of the absorbance of VLP wells to the absorbance of no-VLP wells are reported.

To further understand the binding interactions of aptamer M6-2 relative to those observed for HBGA and antibody NS14 for SYV VP1 (45, 46), a molecular docking simulation was conducted. Previous molecular docking simulations with norovirus capsids and aptamers simply used RNA webservers for the creation of the ssDNA aptamer prior to molecular docking simulations (22). In this study, we employed a method in which, after ssDNA aptamer PDB file construction, a long (>100-ns) MD simulation (with molecular water and counterions) was performed. This allowed us to obtain ssDNA aptamer conformations that more accurately described the aptamer’s conformation in solution. Furthermore, we extracted representative PDB files of the aptamer each 10 ns of the simulation after equilibration was reached (as monitored by the RMSD and radius of gyration) and uploaded these as an ensemble of conformations for molecular docking simulations. A previously performed motif analysis was also used to identify a general interacting region of the ssDNA aptamer (Fig. 4A) (19). An earlier study reported docking between an ssDNA aptamer and the structure of the entire norovirus VP1 protein (22). This approach resulted in models that would not likely occur with an intact capsid, as the interactions are dominated by S-domain residues that are inaccessible in the context of the capsid in its native conformation. However, we limited our study to residues that would be accessible in an assembled capsid. The molecular docking simulations revealed a potential unique interaction surface with the ssDNA aptamer M6-2 compared to HBGA and antibody NS14 (45, 46) (Fig. 4B orange spheres versus magenta and cyan, respectively). This suggests a novel motif for interaction between noroviruses and the ssDNA aptamer that takes advantage of both N-terminal P1 and P2 domain residues, compared to single C-terminal P1 or P2 subdomain residues as NS14 and HBGA, respectively, are known to use. Nonetheless, M6-2 did bind some residues that were close to HBGA interaction residues and bound in N-terminal P1 and P2 domains as opposed to the more distant C-terminal P1 subdomain that NS14 binds (45). This greater binding proximity may also explain why aptamer M6-2 behaves more closely to HBGA than NS14. Despite binding the parts of the P2 subdomain, which is hypervariable, aptamer M6-2 has been shown to be broadly reactive to multiple human norovirus strains, further supporting its use as a favorable alternative for infectivity discrimination. ELASAs with mutated versions of aptamer M6-2 (Fig. 5; see Fig. S3 in the supplemental material) confirmed the binding predictions.

10.1128/mSphere.00298-16.3 Figure S3  Predicted secondary structures of mutated M6-2 aptamers. Secondary structures of mutated versions of aptamer M6-2 were predicted as previously reported with the online Mfold webserver (http://unafold.rna.albany.edu/). Structures: a, M6-2SA; b, M6-2SB; c, M6-2SC. Download Figure S3, PDF file, 0.2 MB. Copyright © 2016 Moore et al. 2016 Moore et al. This content is distributed under the terms of the Creative Commons Attribution 4.0 International license.

To our knowledge, this is the first study to evaluate nucleic acid aptamers as indicators of virus capsid integrity/functionality. The assays and analyses used are easily adaptable to other targets, as aptamers have been successfully used to detect infectious virus in stool samples (19, 21) with minimal modification. The most recent strain of the pandemic human norovirus genotype that causes the majority of human norovirus cases (GII.4 Sydney) was chosen as the model for this proof-of-concept study. Another study evaluating the ability of aptamer M6-2 to predict the capsid functionality of different human norovirus strains with different HBGA binding patterns is under way. Given that aptamer M6-2 binding is broad and not correlated with the selectivity of HBGA binding and the general structures of all human noroviruses are similar, one would expect aptamer M6-2 to behave in a similar manner with other strains as reported with SYV; however, that work is beyond the scope of this study. Future work could also evaluate different norovirus aptamers, which may well bind to different capsid domains. Also, direct comparison of HBGA, antibody, and/or aptamer binding to cultivable viruses would provide direct correlations between virus infectivity and receptor binding assay results. For instance, a murine norovirus aptamer has been reported (20), so aptamer and carbohydrate receptor binding could be compared to plaque assay results. Additionally, the suitability of aptamers for estimation of infectious norovirus particles subjected to other inactivation treatments (e.g., exposure to free chlorine or alcohols; high pressure; pulsed light) would be an appropriate next step. Its broad reactivity, high degree of stability, simplicity of chemical synthesis and modification, and a potential novel interaction interface make aptamer M6-2 a promising ligand for detection and study of human noroviruses, particularly in those instances in which confirmation of virus infectivity loss is necessary.

## MATERIALS AND METHODS

### VLPs and nucleic acid aptamers.

VLPs consisting of the assembled recombinant norovirus capsid proteins of the most recent epidemic GII.4 strain (GII.4 Sydney; SYV) were generously provided in purified form by R. Atmar (Baylor College of Medicine, Houston, TX). VLPs were stored in buffer at a 3.3-mg/ml concentration at 4°C until use. A biotinylated ssDNA aptamer previously reported to be broadly reactive to human norovirus strains, M6-2, was selected for use in this study. It was generated against the P domain of a GII.4 2006b norovirus strain expressed in *Escherichia coli* (19). Additionally, mutated versions of aptamer M6-2 (M6-2SA, M6-2SB, and M6-2SC) with the suspected binding region of the aptamer mutated were also obtained (Table 2). The aptamers were obtained with a 5′ biotin tag in high-performance liquid chromatography (HPLC)-purified form from Integrated DNA Technologies (IDT; Coralville, IA).

**TABLE 2:** Aptamer sequences used in this study^a^

Aptamer	Sequence (5′ → 3′)	Reference
M6-2	AGTATACGTATTACCTGCAGCTGGGAAGAGGTCCGGTAAATGCAGGGTCAGCCCGGAGAGCGATATCTCGGAGATCTTGC	19
M6-2SA	AGTATACGTATTACCGCGAGGGAATGTGACGCAGTCGGAGTTGAACGTCAGCCCGGAGAGCGATATCTCGGAGATCTTGC	This work
M6-2SB	AGTATACGTATTACCTGCAGCTGGGAAGAGTCTTCCCAGCTGCAGGGTCAGCCCGGAGAGCGATATCTCGGAGATCTTGC	This work
M6-2SC	AGTATACGTATTATTTTTTTTTTTTTTTTTTTTTTTTTTTTTTTTTGTCAGCCCGGAGAGCGATATCTCGGAGATCTTGC	This work

a Broadly reactive aptamer M6-2, previously reported to be generated against human norovirus GII.4, was selected for this study. Confirmation of the predicted binding region of the aptamer was conducted by mutation analysis by scrambling the binding region (M6-2SA), closing the region off in a hairpin (M6-2SB), or replacing the binding region with thymine bases (M6-2SC). All aptamers were obtained with a 5′ biotin label in HPLC-purified form from IDT (Coralville, IA).

### Heat treatment of VLPs.

Heat treatment experiments were performed with VLPs diluted to 50 µg/ml in 1× phosphate-buffered saline (PBS; pH 7.2) (for plate assays and DLS) or 10 mM HEPES (pH 7.4) (for TEM). Samples were placed in 13.5-µl aliquots in 0.2-ml Eppendorf tubes and heated with a T100 Thermal Cycler (Bio-Rad, Hercules, CA) at different temperatures (60, 65, 70, 75, or 80°C) for 1 min and at selected temperatures (65 and 68°C) for 2.5 to 100 min. VLPs were treated at 80°C for 5 min (completely denatured) or left untreated for use as negative and positive controls, respectively. A no-VLP PBS negative control was also included for comparison with the completely denatured negative control. Immediately after heat treatment, samples were placed in a DNA Engine (PTC-200) Peltier Thermal Cycler (MJ Research, Hercules, CA) running at 4°C for 5 min to cool. For all plate assays, VLP aliquots were briefly centrifuged and diluted in 1× PBS to 3 µg/ml. One-hundred-microliter volumes of the VLP suspensions were then applied to Costar 3591 medium-binding polystyrene 96-well plates (Fisher, Pittsburgh, PA) overnight at 4°C with light shaking with an orbital shaker prior to assay.

### ELASA.

Aptamer binding to treated VLPs was probed with a previously established assay (21, 47). Briefly, the wells containing the VLPs or PBS (negative control) were blocked with 200 µl of 5% skim milk in PBS-Tween 20 (0.05%, vol/vol; PBST) with a 10 nM mixture of unrelated PCR primers (*Listeria monocytogenes* primers hlyQF/R and L23SQF/R) (48) for 2 h at room temperature with shaking. The plates were washed thrice with 200 µl of PBST and then incubated with 100 µl of 1 μM biotinylated M6-2 aptamer for 1 h. Plates were washed four times with PBST and incubated at 100 µl/well with a 1-mg/ml streptavidin-HRP solution diluted 1:5,000 (vol/vol; Invitrogen, Carlsbad, CA) in PBS for 15 min. Residual conjugate was removed with three washing steps with PBST, and the plate signal was developed with the 3,3′,5,5′-tetramethylbenzidine (TMB) microwell peroxidase substrate system (KPL, Gaithersburg, MD) at 100 µl/well in accordance with the manufacturer’s instructions. The signal was allowed to develop for 2 to 7 min before reactions were stopped by the addition of TMB stop solution (KPL). The absorbance at 450 nm was then recorded with a Tecan Infinite M200pro microplate reader (Tecan Group Ltd., Männedorf, Switzerland).

### ELISA-like HBGA binding assay.

Binding of treated VLPs to HBGAs was simultaneously observed on the same 96-well polystyrene plate as used for aptamers. The HBGA binding assay has been previously reported (30) and resembles the ELASA with minor modifications. Specifically, the wells were washed twice with PBST after blocking and incubated with 30 µg/ml biotinylated blood type A HBGA (catalog no. 01-032; GlycoTech, Gaithersburg, MD) in 100 µl of blocking buffer for 1 h. The signal was developed for 10 to 20 min before the reaction was stopped and absorbance at 450 nm was read.

### ELISA.

This method was also performed as previously reported (49, 50), with slight modification to make it similar to the ELASA and HBGA binding assays. Blocked wells were incubated with 100 µl containing 0.137 µg of NS14 antibody (kindly provided by R. Atmar, Baylor College of Medicine, Houston, TX) (16) in 0.1% skim milk–PBST for 1 h. After washing, the wells were incubated with 0.1 µg of HRP-conjugated goat anti-mouse antibody (catalog no. 62-6520; Invitrogen) in 100 µl of 0.1% skim milk for 1 h. Wells were washed thrice with PBST, and the signal was developed as described above with the TMB substrate system for 1 to 5 min before reactions were stopped and absorbance was read.

### Data analysis.

For plate data analysis, absorbances of negative-control wells for each ligand seeded with completely heat-denatured VLPs (80°C, 5 min) were subtracted from sample well absorbances to account for the nonspecific and target primary-sequence-based ligand interaction. Negative-control-adjusted absorbances of the heat-treated samples were then taken as a percentage of the negative-control-adjusted absorbances of untreated positive control VLPs. At least two wells per temperature and time point were analyzed per assay, and each experiment was replicated three times. Exponential decay rates were calculated with the WYSIWYG 2D plotting tool Grace (http://plasma-gate.weizmann.ac.il/Grace/) with the equation *y* = *a* × *b^x^*, where *y* is the percentage of the positive-control signal, *x* is the time of treatment, and *b* is the rate of decay.

To estimate the apparent percentage of the signal attributable to VLP sequence-dependent versus conformation-dependent binding for all three ligands, absorbance values of VLPs (untreated and heat treated) were adjusted by subtraction of the no-VLP well (accounting for signal due to the plate apparatus itself). The percentage of the signal attributable to the completely denatured capsid (sequence-dependent binding) was then calculated as the ratio of heat-treated absorbance to positive-control wells multiplied by 100. The apparent percentages of ligand binding to the completely denatured capsid for each replicate plate were then averaged, and their standard deviations were determined with Microsoft Excel 2013 (Microsoft, Redmond, WA). Statistical analysis of differences between values derived from plate data was performed by one-way analysis of variance with Tukey’s *post hoc* test with JMP Pro 12 software (SAS, Cary, NC), and *P* values of <0.05 were considered statistically significant.

### DLS.

For temperature dependence data, VLPs that were heat treated for 1 min at 50 µg/ml were rapidly cooled and diluted to 5 µg/ml in 1× PBS and loaded into small-volume (40- to 70-µl) cuvettes at room temperature. Each sample was run in triplicate with a Zetasizer Nano ZSP (Malvern Instruments, Worcestershire, United Kingdom) equipped with a 10-mW HeNe laser at 633 nm and a photodiode located 173º from the incident laser beam. Zetasizer software calculated a Z-average particle diameter with a cumulants fit for each sample from light scattering intensity data. The average of three measurements for each of two samples at each temperature was recorded. For kinetic data, VLPs at 5 µg/ml in 1× PBS were degassed for 10 s in a tabletop centrifuge to prevent bubble formation during measurements and then loaded into a quartz cuvette. After the Zetasizer Nano ZSP reached 68°C, the cuvette was placed inside the sample chamber and allowed to equilibrate for 10 s before the first measurement was started. Zetasizer software calculated the diameters of multiple distinct particle sizes by using a distribution fit for each sample, and the intensity distribution size data were reported for each of three replicate samples.

### TEM.

TEM was used to confirm VLP degradation and observe morphological changes. VLPs at a concentration of 50 µg/ml suspended in 10 mM HEPES (pH 7.4) were heat treated and cooled as described above and then applied to carbon-coated nickel grids (Ladd Research, Williston, VT) for 10 min. Samples were stained with 2% uranyl acetate (SPI Supplies, West Chester, PA) for 45 s, dried in a desiccator, and viewed with a JEOL 1210 transmission electron microscope (JEOL-USA Inc., Peabody, MA) at 80 kV and a magnification of ×50,000.

### Aptamer structure construction.

The secondary structure of aptamer M6-2 and subsequent motif analysis previously reported (19) with the Mfold online server with 0.5 mM magnesium, 1 mM sodium, and 23°C as input parameters (51) were consulted to inform the constraints used in the subsequent docking and structure construction. The M6-2 sequence was converted into RNA, and the MC-Fold and MC-Sym webserver (http://www.major.iric.ca/MC-Fold/) was used to model a three-dimensional structure in PDB file format. Structural constraints (loops and base pairing) identified by the Mfold webserver were used to build a relevant PDB file for the above sequence. The final three-dimensional (3D) RNA-generated PDB file was manually converted to DNA format. This was then subjected to MD simulation for equilibration as described below.

### SYV VP1 PDB file construction.

MODELLER v10.1 (52) was used to construct a model of SYV VP1 by using the crystal structure analysis of the Norwalk virus capsid as the template (PDB entry 1IHM; see Fig. S4 in the supplemental material) (27). During the model-building process, we employed an optimization method involving conjugate gradients and MD simulation to minimize violations of the spatial restraints. Five hundred models were generated from an alignment of SYV VP1 and 1IHM (see Fig. S4) and scored by the internal MODELLER scoring method DOPE (discrete optimized protein energy) (53). DOPE is a statistical potential used to assess homology models in protein structure prediction. DOPE is based on an improved reference state that corresponds to noninteracting atoms in a homogeneous sphere with the radius dependent on a sample native structure; it thus accounts for the finite and spherical shape of the native structures. The structure with the lowest DOPE score was subsequently run through PROCHECK and WHATCHECK (stereochemical quality of a protein structure) for quality (54, 55). Only the highest-quality DOPE score was used for this study.

10.1128/mSphere.00298-16.4 Figure S4  Alignment of 1IHM and SYV capsid sequences. The amino acid sequences of 1IHM (template structure used for SYV VP1 model creation) and SYV VP1 with secondary structure elements of SYV VP1 presented on top (helices with squiggles, β-strands with arrows). Sequence identity is shown by boxing residues in black, similar identity is shown by boxing residues in gray, and gaps are represented by periods. Download Figure S4, PDF file, 0.5 MB. Copyright © 2016 Moore et al. 2016 Moore et al. This content is distributed under the terms of the Creative Commons Attribution 4.0 International license.

10.1128/mSphere.00298-16.5 Figure S5  Cluster analysis of M6-2 and SYV VP1 binding. VP1 dimers are shown in cartoon format. The S domain is gray, the P1 domain is yellow, and the P2 domain is red. Panels: A, cluster 1; B, cluster 2; C, cluster3; D, cluster 4. Clustering of the structures is based on the RMSD of the positions of the Cα and P atoms. Panels C and D denote unrealistic binding modes, as the S domain further interacts with other S-domain dimers to form the capsid. These depict orientations in which the aptamer would interfere with capsid formation. Panels A and B represent possible modes of interaction of SYV and a DNA aptamer. Download Figure S5, PDF file, 0.3 MB. Copyright © 2016 Moore et al. 2016 Moore et al. This content is distributed under the terms of the Creative Commons Attribution 4.0 International license.

### MD simulations.

MD simulations were performed to provide a better representation of the DNA aptamer’s conformational flexibility and to more accurately characterize the aptamer’s solution structure for future molecular docking experiments. MD simulations were performed with the GROMACS 4.4.5 software package by using the AMBER 99sb-ildn force field and the flexible simple point-charge water model (56). The initial structures were immersed in a periodic water box with a dodecahedron shape and neutralized with counterions. Electrostatic energy was calculated by the particle mesh Ewald method. The cutoff distance for the calculation of the Coulomb and van der Waals interactions was 1.0 nm. After energy minimization by a steepest-descent method, the system was subjected to equilibration at 300 K and normal pressure for 100 ps under the conditions of position restraints for heavy atoms and LINCS constraints for all bonds. The system was coupled to the external bath by Parrinello-Rahman pressure and temperature coupling. The final MD calculations were performed under the same conditions, except that the position restraints were removed and the simulation was run for 100 ns. MD simulations were performed on the DNA aptamer, as well as the MODELLER-constructed PDB file of SYV VP1. A final PDB file of each 10-ns step in the 100-ns simulation was constructed and further used in HADDOCK molecular docking with VLPs. All images were produced with PyMOL (The PyMOL Molecular Graphics System, version 1.7.4; Schrödinger, LLC).

### SYV VP1-aptamer modeling with HADDOCK.

Default HADDOCK (high-ambiguity-driven docking) parameters were used throughout all docking procedures (57, 58). The docking procedure consisted of monomer SYV VP1 model PDB file constructed as stated above. Active residues (residues directly involved in the interaction) within SYV VP1 were defined as those with >50% solvent accessibility (calculated by Naccess) (28). No residues within the S domain were defined as active, as the aptamer was generated against only the P domain, and none of the residues within the dimerization interface within the P1/P2 domain were defined as active residues. No protein passive residues (those residues indirectly involved in the interaction) were defined. Active bases within the DNA aptamer were defined as bases 14 to 45, while passive residues were defined as bases 1 to 13 and 46 to 81. One thousand structures were generated within the first rigid docking iteration, the 20% with the lowest HADDOCK scores were then further refined in a semiflexible *in vacuo* environment, and all structures from the semiflexible iteration were further water refined in the final iteration. Each docking attempt was performed 10 times, and the solution with the lowest HADDOCK score was retained. The RMSDs of the complexes were calculated with the McLachlan algorithm (59) as implemented in ProFit (A. C. R. Martin; http://www.bioinf.org.uk/software/profit/). A cluster analysis of the final docking solutions was performed with a minimum cluster size of five. The cutoff for clustering was manually determined for each docking run. The RMSD matrix was calculated over the backbone atoms (C, N, HN, Cα, and P atoms) of the interfacing residues. All images were produced with PyMOL.

### ELASA confirmation of docking prediction.

To confirm docking predictions, versions of M6-2 with the predicted docking region mutated in three different ways were obtained. The suspected binding region was randomly scrambled (M6-2SA), closed off as a hairpin (M6-2SB), or replaced with thymine (M6-2SC) (Table 2; see Fig. S3 in the supplemental material). ELASAs with various aptamer concentrations (0.001 to 10 µM) were conducted for these aptamers along with M6-2 as reported above. Simple binding analysis of the VLP/no-VLP ratio is reported as described previously (19, 21), where the absorbance of wells containing SYV VLPs was divided by the absorbance of wells without the VLPs.
