# Supplementary figures and images for: Human Norovirus Aptamer Exhibits High Degree of Target Conformation-Dependent Binding Similar to That of Receptors and Discriminates Particle Functionality
Source: mSphere. 2016 Nov 2;1(6):e00298-16. doi: 10.1128/mSphere.00298-16 (PMC5093151; doi:10.1128/mSphere.00298-16)

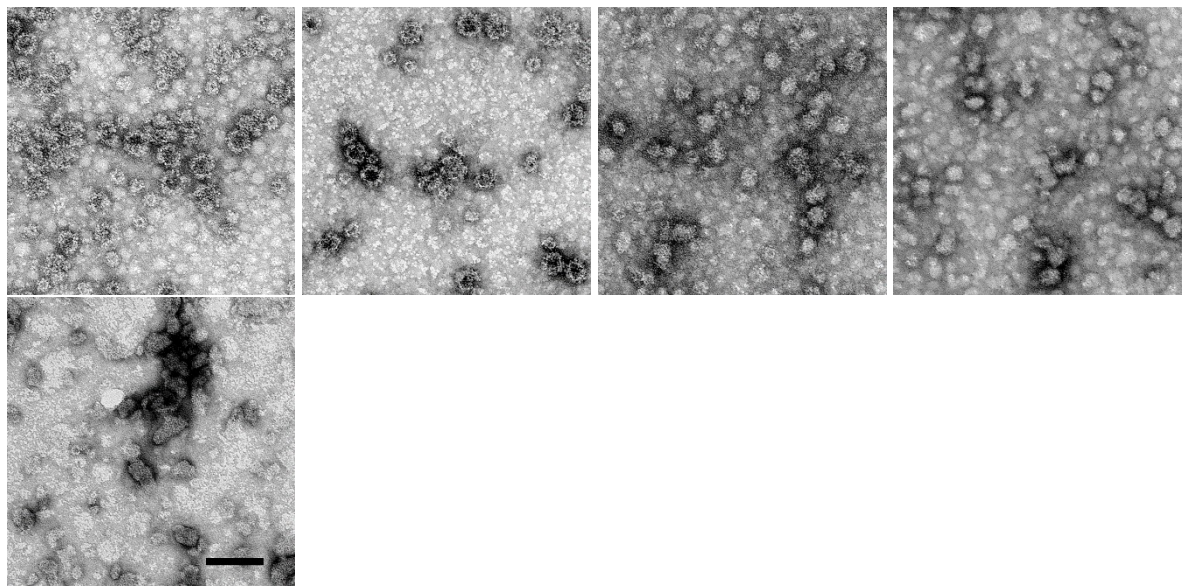

Figure S1

Supplement: Figure S1 [file sph006162179sf1.pdf]

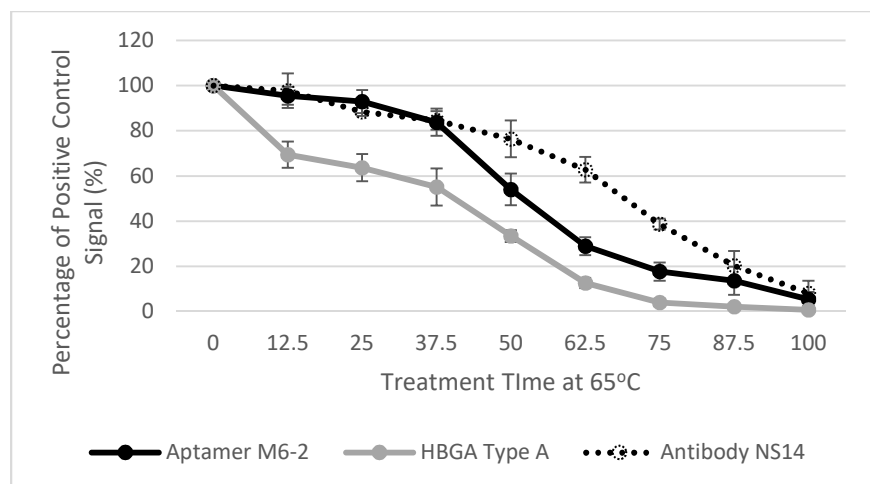

Figure S2

Supplement: Figure S2 [file sph006162179sf2.pdf]

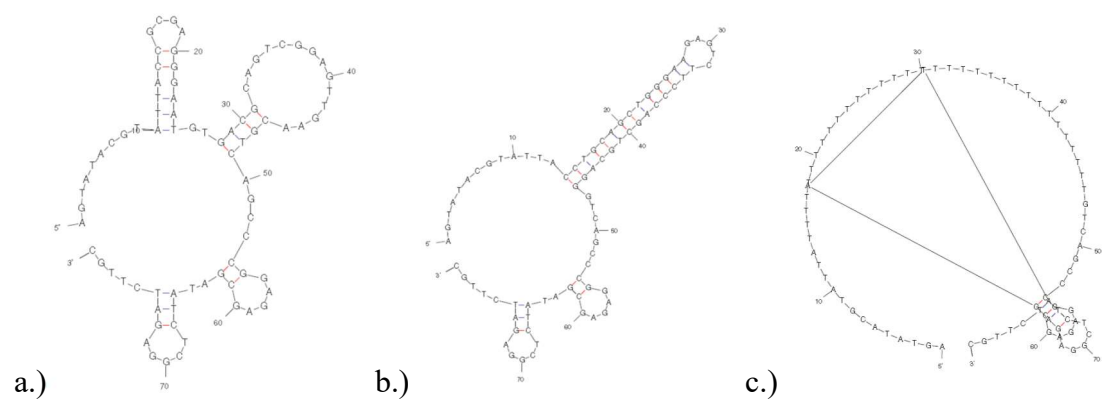

Figure S3

Supplement: Figure S3 [file sph006162179sf3.pdf]

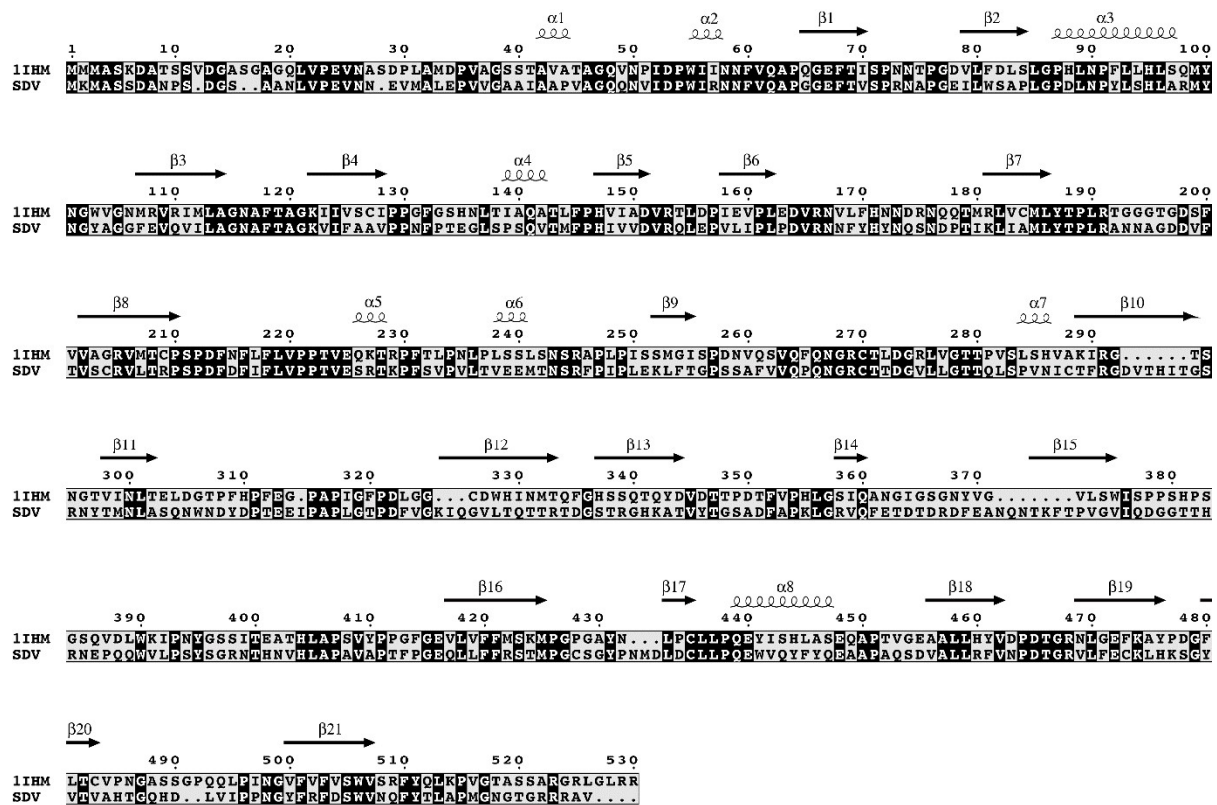

Figure S4

Supplement: Figure S4 [file sph006162179sf4.pdf]

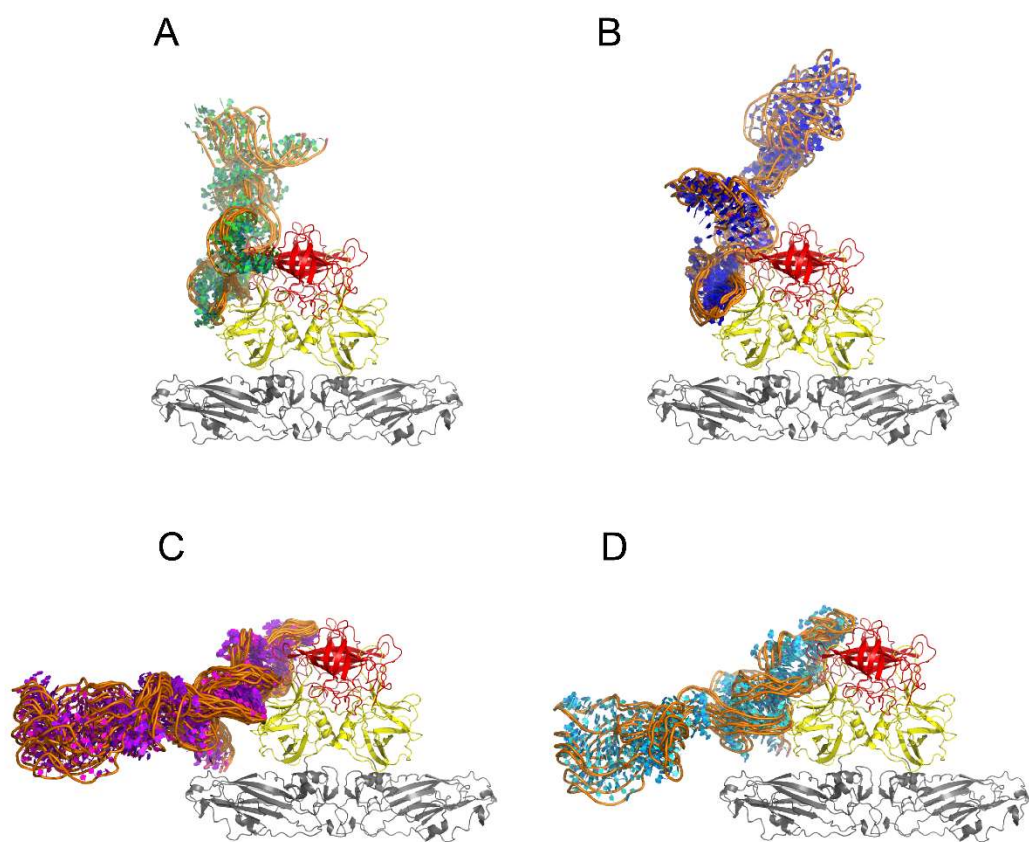

Figure S5

Supplement: Figure S5 [file sph006162179sf5.pdf]
